# CRISPLD2 Is a Target of Progesterone Receptor and Its Expression Is Decreased in Women with Endometriosis

**DOI:** 10.1371/journal.pone.0100481

**Published:** 2014-06-23

**Authors:** Jung-Yoon Yoo, Heesung Shin, Tae Hoon Kim, Won-Seok Choi, Susan D. Ferguson, Asgerally T. Fazleabas, Steven L. Young, Bruce A. Lessey, Un-Hwan Ha, Jae-Wook Jeong

**Affiliations:** 1 Department of Obstetrics, Gynecology & Reproductive Biology, Michigan State University, College of Human Medicine, Grand Rapids, Michigan, United States of America; 2 Department of Biotechnology and Bioinformatics, Korea University, Sejong, South Korea; 3 Department of Food Science and Technology, Korea National University of Transportation, Chungbuk, South Korea; 4 Department of Obstetrics and Gynecology, University of North Carolina, Chapel Hill, North Carolina, United States of America; 5 Department of Obstetrics and Gynecology, University Medical Group, Greenville Hospital System, Greenville, South Carolina, United States of America; The University of Georgia, United States of America

## Abstract

Endometriosis, defined as the presence of endometrial cells outside of the uterine cavity, is a major cause of infertility and pelvic pain, afflicting more than 10% of reproductive age women. Endometriosis is a chronic inflammatory disease and lipopolysaccharide promotes the proliferation and invasion of endometriotic stromal cells. Cysteine-rich secretory protein LCCL domain-containing 2 (CRISPLD2) has high affinity for lipopolysaccharide and plays a critical role in defense against endotoxin shock. However, the function of CRISPLD2 has not been studied in endometriosis and uterine biology. Herein, we examined the expression of CRISPLD2 in endometrium from patients with and without endometriosis using immunohistochemistry. The expression of CRISPLD2 was higher in the secretory phase in human menstrual cycle compared to proliferative phase. The expression of CRISPLD2 was significantly decreased in the endometrium of women with endometriosis in the early secretory phase compared to women without endometriosis. The increase of CRISPLD2 expression at the early secretory and dysregulation of its expression in endometriosis suggest progesterone (P4) regulation of CRISPLD2. To investigate whether CRISPLD2 is regulated by P4, we examined the expression of the CRISPLD2 in the uteri of wild-type and progesterone receptor knock out (PRKO) mice. The expression of CRISPLD2 was significantly increased after P4 treatment in the wild-type mice. However, CRISPLD2 expression was significantly decreased in the (PRKO) mice treated with P4. During early pregnancy, the expression of CRISPLD2 was increased in decidua of implantation and post-implantation stages. CRISPLD2 levels were also increased in cultured human endometrial stromal cells during *in vitro* decidualization. These results suggest that the CRISPLD2 is a target of the progesterone receptor and may play an important role in pathogenesis of endometriosis.

## Introduction

Endometriosis is one of the most common causes of chronic pelvic pain and infertility. It affects 10% of women of reproductive age and the incidence increases to 35–50% in infertile women [1], [2]. The initiation of endometriosis is difficult to evaluate because the disease has usually been prevalent for 8–11 years at the time of clinical diagnosis [2], [3]. Surgical removal of lesions and hormonal suppression are the current gold standards of therapy, but both approaches are associated with various side effects and a high incidence of relapse. Endometriosis is a multifactorial disease [4]. One of the major hallmarks of these uterine diseases is disruption of steroid hormone control of uterine cell proliferation and differentiation [5]. Elucidating the molecular mechanisms by which the steroid hormones control uterine physiology is paramount to understanding the pathology of these diseases.

The uterine endometrium is comprised of epithelial and stromal compartments that undergo dynamic molecular and morphological changes to allow for embryo implantation and development. These changes are mediated by the ovarian steroids estrogen (E2) and progesterone (P4). E2 stimulates proliferation of uterine epithelial cells while P4 is inhibitory to E2-mediated proliferation of the epithelium [6], [7]. P4 achieves this inhibition of proliferation through coordinating stromal-epithelial cross-talk. Disruption of the steroidal control of uterine proliferation leads to the pathology found in endometriosis. P4 exposure is a negative risk factor for endometriosis [8], and pregnancy or progestin-based therapies can lead to disease regression in some women [9], [10]. However a portion of patients with endometriosis and pelvic pain do not respond to treatment with progestins. Moreover, P4-induced molecular changes in the eutopic (intrauterine) endometrial tissue of women with endometriosis are either blunted or undetectable. These *in vivo* observations are indicative of resistance to P4 action in endometriosis [11]. Published microarray gene expression profiles of the endometrium of women with or without endometriosis showed that a number of P4 target genes were dysregulated during the window of implantation, at which time the endometrium is exposed to the highest levels of P4 [5], [12]. Therefore, understanding the role of the progesterone receptor and its target genes is important for successful therapies in endometriosis [13], [14].

The cysteine-rich secretory protein LCCL (Limulus factor C, cochlear protein Coch-5b2 and late gestation lung protein Lgl1) domain-containing 2 (*CRISPLD2*) gene is a member of the cysteine-rich secretory protein, antigen 5, and pathogenesis-related 1 proteins (CAP) protein superfamily and is 91 kb, located on chromosome 16q24.1, and is consists of 15 exons [15]. It is reported that *CRISPLD2* is significantly associated with craniofacial morphogenesis [16], [17] as well as in alveolar development and branching morphogenesis [18], [19]. *Crispld2* knockout (KO) mice are embryonic lethal at 9.5 dpc. Heterozygous *Crispld2* mice display abnormal lung development with delayed alveolar maturation [20]. *Crispld2* plays a role in development or differentiation in the lung and face [16]–[19]. Affinity of the LCCL domains to lipid A of endotoxin such as lipopolysaccharide (LPS) can block its interaction with host endotoxin receptors [21]–[23]. Therefore, *CRISPLD2* was suggested as an anti-inflammatory response gene [21].

During decidualization, P4-mediated differentiation is induced along with an inflammatory response [13], [24]. P4 normally mediates the balance between anti-inflammatory and proinflammatory processes in uterus. However, progesterone resistance is observed by alterations in progesterone responsive gene and protein expression in women with endometriosis. Pelvic endometriosis lesions are thought to derive predominantly from retrograde menstruation, which occurs in nearly all women [25], [26]. A key difference in women with endometriosis appears to be that retrograde menstruation alters the inflammatory response and immunosurveillance within the peritoneal fluid, facilitating proliferation and recurrence of new disease [27]. In affected women, inflammatory responses are seen both systemically and in eutopic endometrium, which is populated by inflammatory leukocytes. It is well recognized that chronic inflammation is associated with endometriosis as with the majority of human diseases including cancers [28]. Endometriosis is an estrogen dependent inflammatory condition. However, the function of *CRISPLD2* has not been studied in endometrium and endometriosis. In the present study, we investigated the expression level of CRISPLD2 in endometrium from patients with and without endometriosis. We also explored the spatiotemporal expression and regulation of CRISPLD2 in the response to P4-PR as well as during early pregnancy of mouse.

## Materials and Methods

### Ethics statement

The study has been approved by Institutional Review Committee of Michigan State University (Grand Rapids, MI), Greenville Health System (Greenville, SC) and University of North Carolina (Chapel Hill, NC), and written informed consent was obtained from all participants.

### Human endometrium samples

The human endometrial samples were collected from the Michigan State University's Center for Women's Health Research Female Reproductive Tract Biorepository, the Greenville Hospital System, and the University of North Carolina. For experiments examining CRISPLD2 expression in endometrium throughout the menstrual cycle, endometrium was obtained at the time of laparoscopic surgery from 26 regularly cycling women between the age of 18 and 50 undergoing hysterectomy for benign indications (i.e. fibroids, prolapse). The presence or absence of disease was confirmed during surgery. Women laparoscopically negative for this disease were placed into the control group, whereas women laparoscopically positive for this disease were placed in the endometriosis group. Use of an IUD or hormonal therapies in the 3 months preceding surgery was exclusionary for this study. Histologic dating of endometrial samples was done based on the criteria of Noyes [29] and confirmed by subsequent histo-pathological examination by an experienced Fertility specialist (B.A.L.). To compare CRISPLD2 expression patterns of endometrium from early secretory phase women with and without endometriosis, 17 samples were collected from eutopic (n = 12) and ectopic (n = 5) endometrium of women with endometriosis and 7 samples were from women without endometriosis.

### Primary human endometrial stromal cell cultures and *in vitro* decidualization

Human endometrial stromal cells (hESCs) were isolated from endometrial tissue by collagenase digestion as previously described [30], [31]. hESCs were maintained in phenol red–free RPMI-1640 medium (Gibco, Grand Island, NY) containing 0.1 mM sodium pyruvate (Gibco, Grand Island, NY), 10% fetal bovine serum (FBS; Gibco, Grand Island, NY) depleted of steroids by pre-treatment with dextran-coated charcoal (Sigma Aldrich, St. Louis, MO) (Charcoal-stripped FBS; CS-FBS), and 1% penicillin streptomycin (P/S; Gibco, Grand Island, NY). To induce *in vitro* decidualization, hESCs were transferred to OPTI-MEM medium (Gibco, Grand Island, NY) containing 2% CS-FBS, 10 nM estradiol (E2, Sigma-Aldrich, St. Louis, MO), 1 mM medroxyprogesterone acetate (MPA; Sigma-Aldrich, St. Louis, MO), 50 µM cAMP (Sigma-Aldrich, St. Louis, MO), and 1% P/S. Decidualization medium was changed every two days and treatment lasted for 6 days. All results from *in vitro* decidualization were confirmed in hESCs obtained from at least three independent biological replicates.

### Animals and tissue collection

Mice were cared for and used in the designated animal care facility according to the Michigan State University institutional guidelines. All animal procedures were approved by the Institutional Animal Care and Use Committee of Michigan State University. For the early pregnancy study, wild-type C57BL/6 mice at 8 weeks of age were mated with wild-type C57BL/6 male mice and uterine samples from pregnant mice were obtained at different days of pregnancy. The morning of vaginal plug observation was designated as 0.5 days postcoitus (dpc) (n = 3). For the study of steroid hormone regulation, wild-type and PRKO mice [32] at 6 weeks of age were ovariectomized. Two weeks post-surgery, ovariectomized mice were injected with vehicle (sesame oil; Veh), progesterone (1 µg/mouse; P4), estradiol (0.1 µg/mouse; E2), or E2 plus P4. Mice were euthanized at 6 hour after injection (n = 3 per genotype per treatment). Uterine tissues were immediately frozen at the time of dissection and stored at −80°C for RNA extraction or fixed with 4% (v/v) paraformaldehyde for immunohistochemistry.

### Quantitative real-time PCR

Total RNA was isolated using the RNeasy total RNA isolation kit (Qiagen, Valencia, CA). The expression levels of *CRISPLD2*, *IGFBP1*, and *PRL* mRNA were measured by real-time PCR SYBR Green detection system (Bio-Rad, Hercules, CA) according to the manufacturer's instructions (PE Applied Biosystems, Foster City, CA). mRNA quantities were normalized against the housekeeping gene, 18S RNA. The sequences of the primers used for human *CRISPLD2* were 5′-CGGACGAGATGAATGAGGTG-3′ and 5′-TGACCGCAGAGGTT TTCTTG-3′, mouse *Crispld2* were 5′- CACCGAGAAAAGCCTCACAA-3′ and 5′-CTCGGCA TATGCTGGAAGAA-3′, for *IGFBP1* were 5′- CTATGATGGCTCGAAGGCTC-3′ and 5′-TTC TTGTTGCAGTTTGGCAG-3′, for *PRL* were 5′- CATCAACAGCTGCCACACTT-3′ and 5′-C GTTTGGTTTGCTCCTCAAT-3′, and for *18S* were 5′- GTAACCCGTTGAACCCCATT-3′ and 5′- CCATCCAATCGGTAGTAGCG-3′.

### Immunohistochemistry

Uterine sections from paraffin-embedded tissue were cut at 6 µm and mounted on silane-coated slides, deparaffinized and rehydrated in a graded alcohol series. Sections were preincubated with 10% normal goat serum in PBS (pH 7.5) and then incubated with primary antibody diluted in 10% normal goat serum in PBS (pH 7.5) overnight at 4°C at 1∶2000 dilution for CRISPLD2 (HPA030055; Sigma Aldrich, St. Louis, MO). On the following day, sections were washed in PBS and incubated with 1∶1000 diluted secondary anti-rabbit antibody (BA-1000; Vector Laboratories, Burlingame, CA) for one hour at room temperature. Horseradish peroxidase conjugated streptavidin (SNN1004; Vector Laboratories, Burlingame, CA) at a dilution of 1∶1000 was added to the slides and incubated for 30 min. Immunoreactivity was detected using DAB (SK-4100; Vector Laboratories, Burlingame, CA). Next, the nuclei were stained with hematoxylin (Biocare Medical, Concord, CA) for 30 sec. The slides were subsequently washed in water and increasing gradients of ethanol, then placed in xylene before mounting in a xylene-based mounting solution (Fisher Scientific Inc, Hanover park, IL). The immunostained sections were observed under a microscope.

### Western blot analysis

Cells were lysed using lysis buffer (10 mM Tris-HCl (pH 7.4), 150 mM NaCl, 2.5 mM EDTA, and 0.125% Nonidet P-40 (vol/vol) in distilled water supplemented with both a protease inhibitor cocktail (Roche, Indianapolis, IN) and a phosphatase inhibitor cocktail (Sigma Aldrich, St. Louis, MO). Samples containing 30 µg proteins were applied to 8% SDS-PAGE. The separated proteins were transferred onto a polyvinylidene difluoride membrane (Millipore Corp., Bedford, MA). Membranes were blocked for two hours with 0.5% casein (wt/vol) in PBS with 0.1% Tween 20 (vol/vol) (Sigma Aldrich, St. Louis, MO) and probed with anti-CRISPLD2 (HPA030055; Sigma Aldrich, St. Louis, MO) antibody. Immunoreactivity was visualized by incubation with a horse-radish peroxidase-linked second antibody and treated with ECL reagents. To control for loading, the membrane was stripped, probed with anti-Actin (sc1616; Santa Cruz Biotechnology, Inc., Santa Cruz, CA).

### Statistical analysis

Statistical analyses were performed using one-way ANOVA analysis followed by Tukey's post hoc multiple range test using the Instat package from GraphPad (GraphPad Software, Inc., San Diego, CA). *p*<0.05 was considered statistically significant.

## Results

### Dysregulation of CRISPLD2 expression in endometrium from women with endometriosis

**Figure 1:**
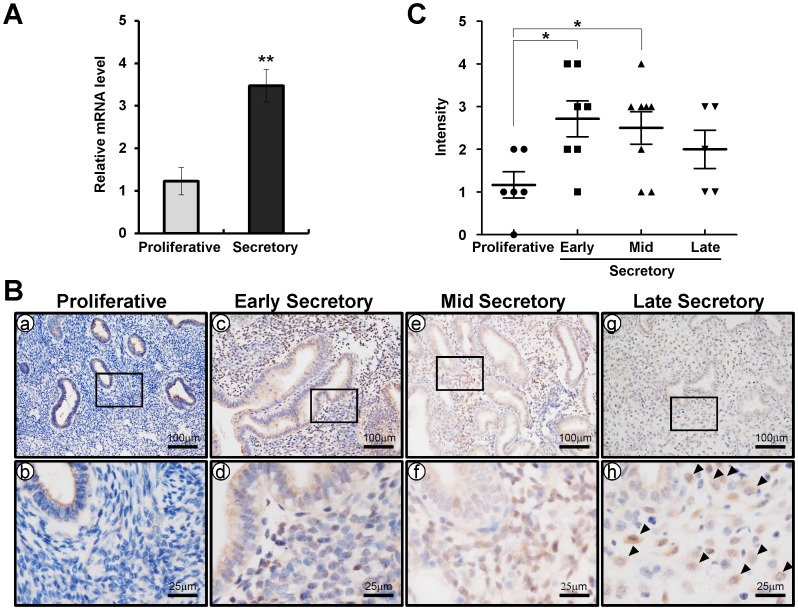
Comparison of CRISPLD2 expression in the endometrium during menstrual cycle. (A) Quantitative real-time PCR analysis of *CRISPLD2* gene expression in proliferative and secretory phase during the menstrual cycle normalized using the ddCt method to the 18S gene. The results represent the mean ± SEM. ** *p*<0.01. (B) Immunohistochemical analysis of CRISPLD2 in proliferative (a and b) and early (c and d), mid (e and f), and late (g and h) secretory phase of the menstrual cycle. Black arrow head indicates a decidualized cell. (C) CRISPLD2 was scored by measuring expression intensity of endometrial cells. The results represent the mean ± SEM. * *p*<0.05.

**Figure 2:**
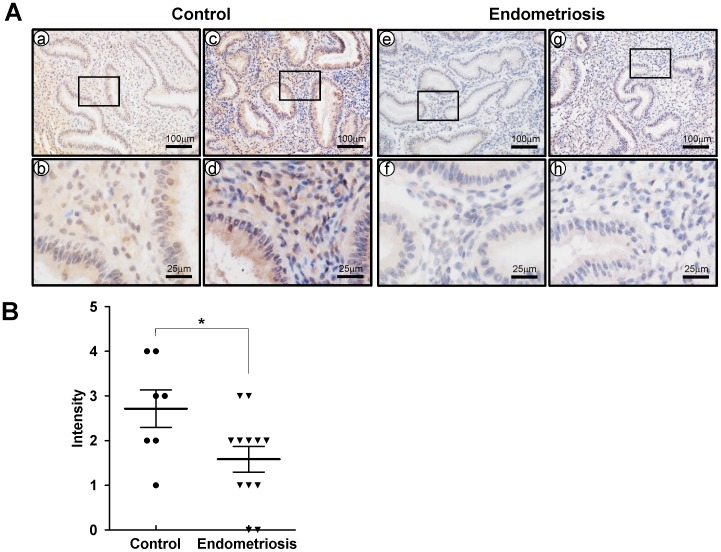
Comparison of CRISPLD2 expression in the endometrium between women with and without diagnosed endometriosis. (A) Immunohistochemical analysis of CRISPLD2 in paired (a - d from women without diagnosed endometriosis; e - h from women with endometriosis) endometrium. (B) CRISPLD2 was scored by measuring expression intensity of endometrial cells. The results represent the mean ± SEM. * *p*<0.05

**Figure 3:**
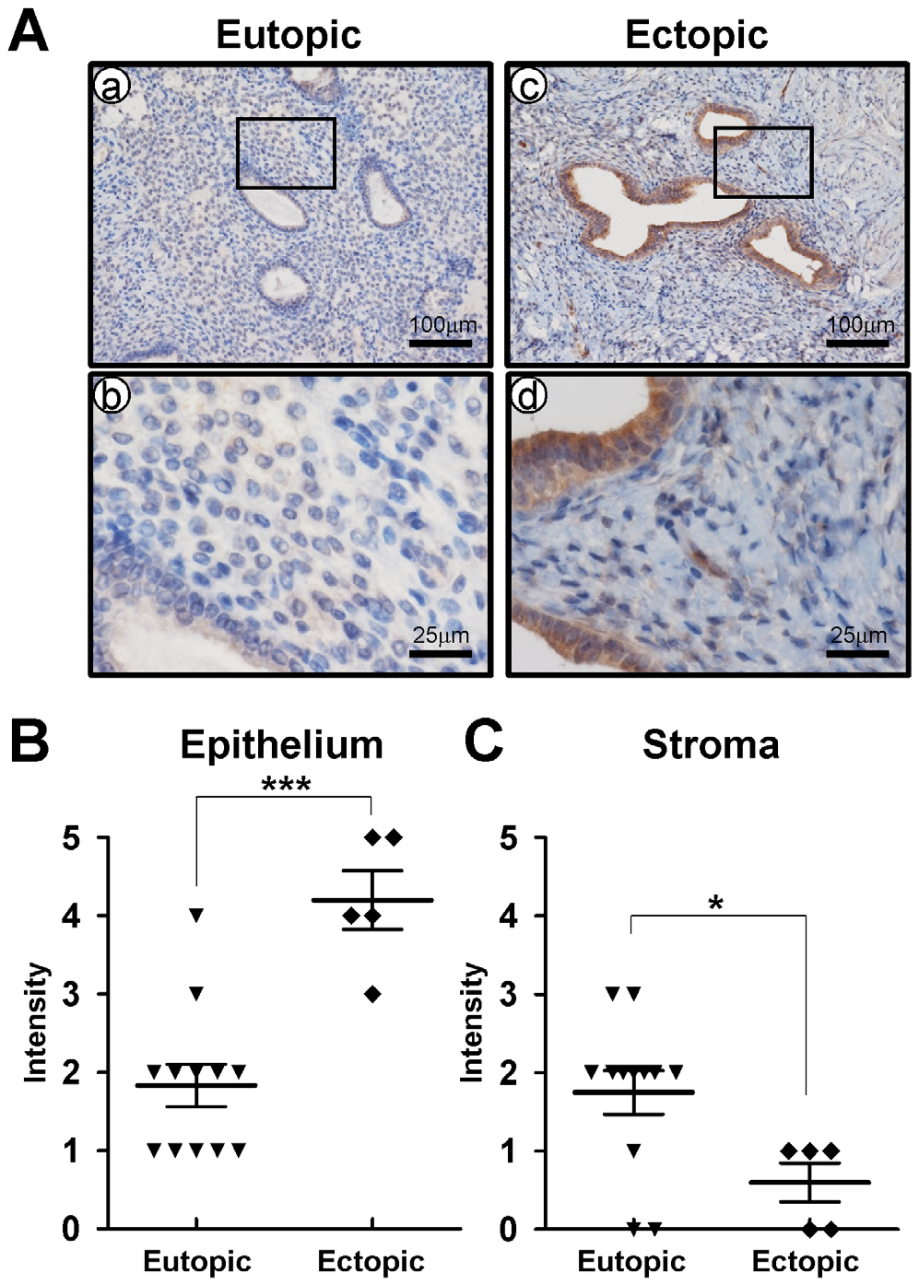
Comparison of CRISPLD2 expression in the endometrium between eutopic and ectopic endometriosis lesions. (A) Representative immunohistochemistry of CRISPLD2 in eutopic and ectopic endometrium with endometriosis. (B and C) CRISPLD2 was scored by measuring expression intensity of endometrial epithelial (B) and stromal (C) cells. The results represent the mean ± SEM. *** *p*<0.001; * *p*<0.05

Quantitative real-time PCR was conducted on endometrial sections from endometrial biopsies to determine expression of CRISPLD2 throughout the endometrium (Fig. 1A). *CRISPLD2* mRNA was significantly increased in the secretory phase (n = 13) as compared to proliferative phase (n = 5). We next used immunohistochemical analysis to examine the cell-specific expression of CRISPLD2 in endometrium from proliferative phase (n = 6) and early (n = 7), mid (n = 8), and late (n = 5) secretory phase in women without endometriosis. CRISPLD2 protein expression was significantly higher in endometrial stromal and epithelial cells of the early and mid secretory phase as compared to proliferative phase (Fig. 1B and C). Interestingly, we observed that decidual cells have strong CRISPLD2 staining in late secretory phase (Fig. 1B g and h). To determine if CRISPLD2 is dysregulated in endometriosis, we examined the expression of CRISPLD2 in endometrium from early secretory phase women with and without endometriosis using immunohistochemistry. The expression levels of CRISPLD2 was scored by measuring expression intensity of early secretory phase women without endometriosis (n = 7) and eutopic secretory phase endometrium from women with endometriosis (n = 12). Interestingly, the protein level of CRISPLD2 was significantly lower in the eutopic endometrium obtained during the secretory phase from women with endometriosis compared to cells from the control endometrium (Fig. 2). It has been shown that in women with endometriosis, gene expression profiles in eutopic endometrium and ectopic endometrium differs, and that alterations in gene expression of the ectopic endometrium play an important role in the pathogenesis of endometriosis. Therefore, we compared the levels of CRISPLD2 protein in eutopic endometrium (n = 12) and ectopic lesions (n = 5) from women with endometriosis. CRISPLD2 protein was significantly higher in the epithelium of ectopic lesions from women with endometriosis as compared to eutopic endometrium (Fig. 3B). However, CRISPLD2 protein expression was very weak in the stromal cells of ectopic lesions (Fig. 3C).

### CRISPLD2 expression in human endometrial stromal cells (hESCs) during *in vitro* decidualization

**Figure 4:**
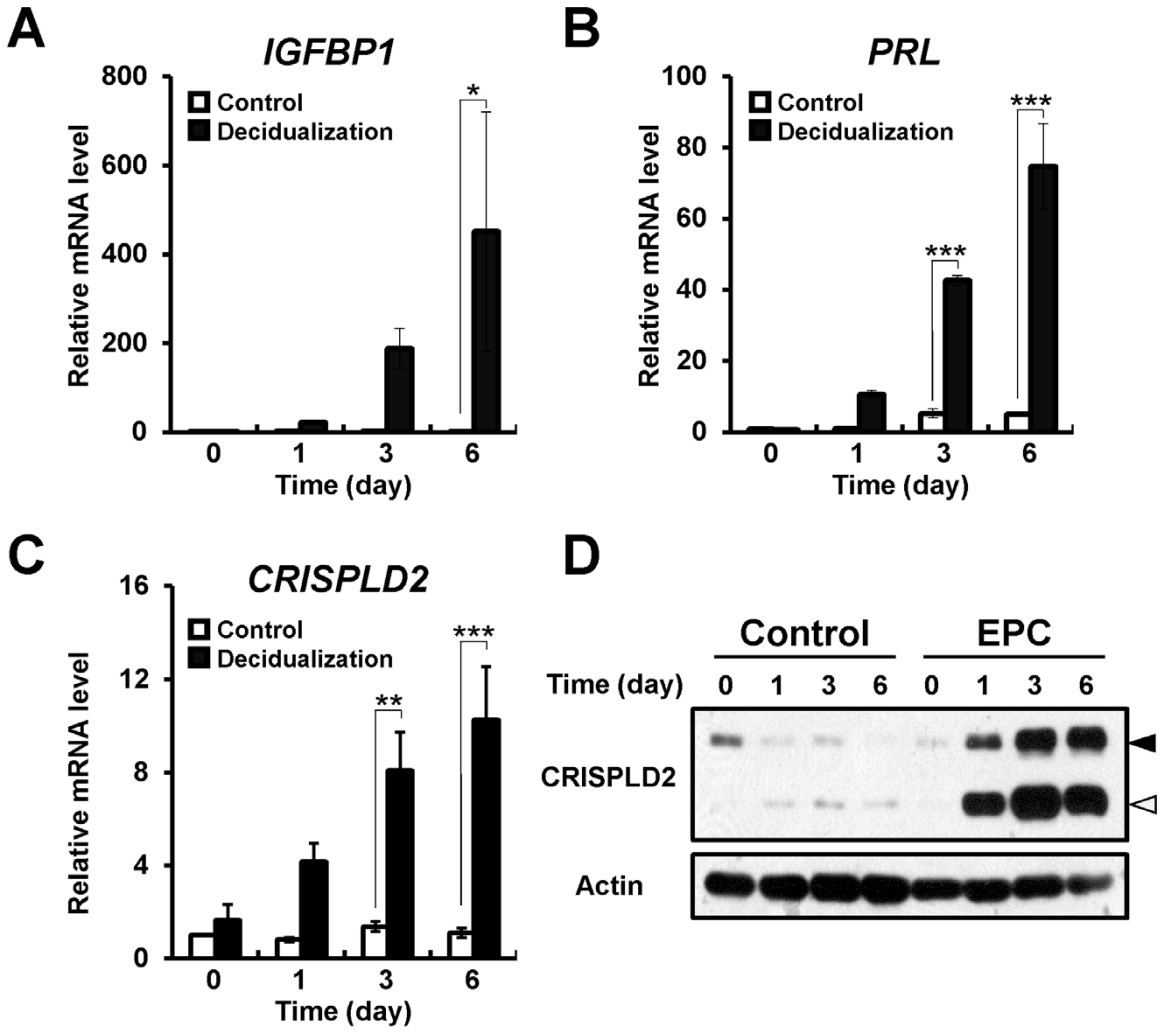
Expression of CRISPLD2 during *in vitro* decidualization of hESCs. (A to C) Expression of decidualization marker genes, *IGFPB1* (A) and *PRL* (B) and *CRISPLD2* gene (C) were examined during *in vitro* decidualization of hESCs. The results represent the mean ± SEM. * *p*<0.05; **, *p*<0.01; ***, *p*<0.001. (D) Protein level of CRISPLD2 was measured by Western blot analysis. Actin was used as loading control. Black arrow head indicates a non-secreted CRISPLD2 and white arrow head indicates secreted CRISPLD2.

P4 is a critical regulator of reproductive events associated with endometrial stromal cell decidualization and maintenance of pregnancy. Eutopic human ESCs from patients with endometriosis showed an impaired decidualization [33], [34]. To examine whether CRISPLD2 is regulated during human endometrial stromal cell differentiation, we used a well characterized *in vitro* model [31] to induce decidualization in cultured human primary endometrial stromal cells (hESCs) following treatment with estrogen, progesterone and cAMP. hESCs possess a fibroblast-like morphology. After *in vitro* decidualization treatment, hESCs enlarged and became round in shape, typical of the decidual transformation (data not shown). Quantitative PCR analysis revealed significantly increased expression levels of the decidualization marker genes (*IGFBP1*and *PRL*) after treatment (Fig. 4A and B). Quantitative PCR analysis and Western blot analysis were conducted on mRNA and proteins from control hESC or hormone-treated hESCs on day 0, 1, 3, and 6 of *in vitro* decidualization. *CRISPLD2* mRNA was significantly increased during *in vitro* decidualization (Fig. 4C). Furthermore, non-secreted and secreted forms of CRISPLD2 proteins were increased on day 1, 3, and 6 of *in vitro* decidualization (Fig. 4D). These results suggest that CRISPLD2 plays an important role in the P4 regulation of human stromal cell decidualization.

### *Crispld2* expression during early pregnancy in mice

**Figure 5:**
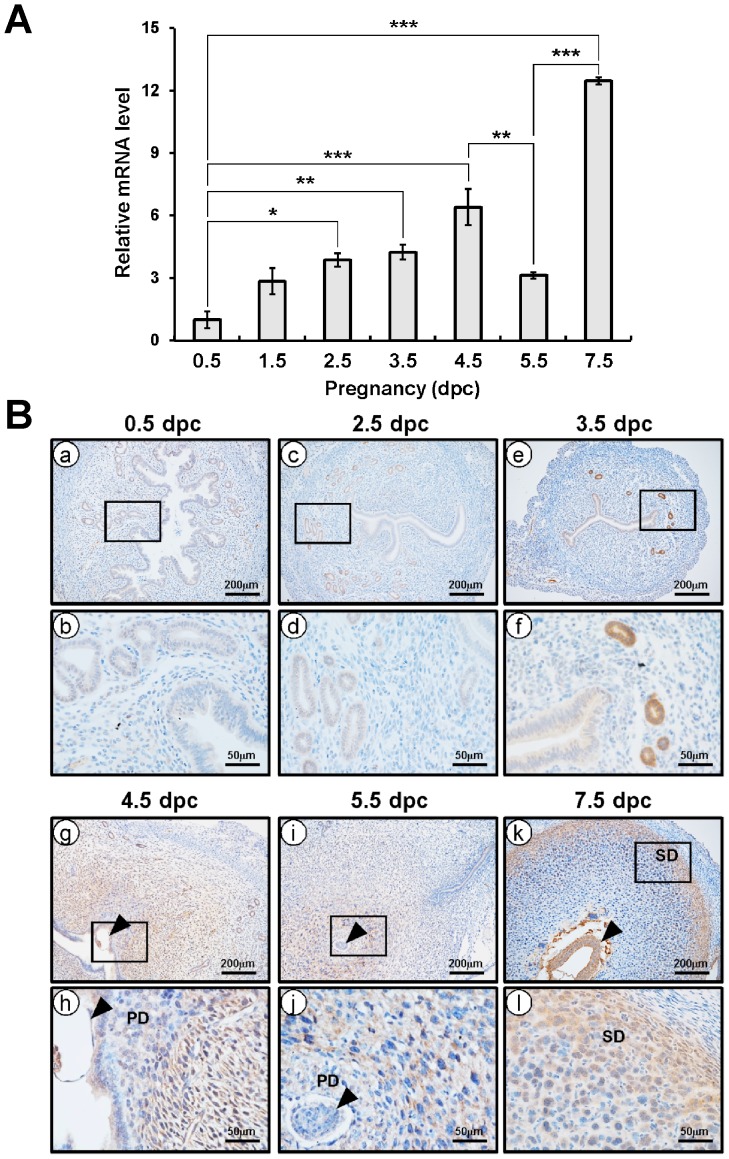
The expression level of *Crispld2* in pregnancy and the localization pattern of CRISPLD2 during early pregnancy. (A) The expression level of *Crispld2* was measured in uteri of early pregnancy. Total RNA used for the quantitative real-time PCR assays was prepared from early pregnancy uteri. The results represent the mean ± SEM of three independent RNA sets. * *p*<0.05; ** *p*<0.01; ***; *p*<0.001. (B) The localization pattern of CRISPLD2 during natural pregnancy by immunohistochemistry were determined at 0.5 dpc (a and b), 2.5 dpc (c and d), 3.5 dpc (e and f), 4.5 dpc (g and h), 5.5 dpc (i and j), and 7.5 dpc (k and l). Black arrow head indicates embryo, PD means primary differentiated decidual cells, and SD means secondary decidual zone.

To investigate the expression of *Crispld2* during early pregnancy, levels of *Crispld2* were examined in uteri of C57BL/6 female mice during early pregnancy. The initiation of pregnancy was marked by the presence of the postcoital vaginal plug (0.5 dpc). As shown in Fig. 5A, the expression of *Crispld2* was detected on 0.5 dpc, which gradually increased until 7.5 dpc, reaching statistical significance after 2.5 dpc in the uterus. Interestingly, the expression of *Crispld2* was significantly decreased on 5.5 dpc compared to 4.5 and 7.5 dpc. To further investigate the spatiotemporal expression profile of CRISPLD2 protein in the pregnant uterus, we performed immunohistochemistry during sequential time points. The expression of CRISPLD2 was not observed at 0.5 dpc. CRISPLD2 was highly expressed in the glandular epithelium but at lower levels in the stromal cells at 3.5 dpc and strongly expressed in the epithelial and non-decidual stromal cells at 4.5 dpc, the period during which the uterine epithelium prepares for and permits the embryo to implant and stromal cells are differentiated to decidual cells. The strong expression of CRISPLD2 was disappeared at 5.5 dpc however, CRISPLD2 was more detected in fully differentiated decidual cells compared with primary differentiated decidual cells. Additionally, CRISPLD2 was highly expressed in the secondary decidual zone (further from the embryo) at 7.5 dpc than in the primary decidual zone (closer to the embryo) (Fig. 5B). However, CRISPLD2 was detected less after 9.5 dpc (data not shown). These results suggest that CRISPLD2 have an important role for implantation and decidualization during early pregnancy.

### *Crispld2* is a progesterone and PR target gene in the murine uterus

**Figure 6:**
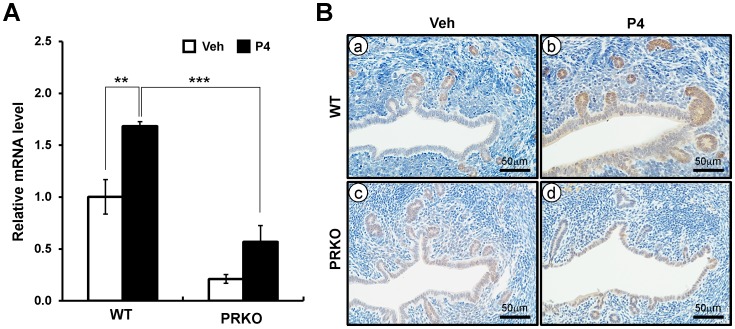
The expression of CRISPLD2 in wild-type or PRKO mice. (A) The expression level of *Crispld2* from P4 treated wild-type or PRKO uteri by quantitative real-time PCR. Total RNA used for the quantitative real-time PCR was prepared from wild-type or PRKO uteri treated with P4 or vehicle for 6 hours. The results represent the mean ± SEM of three independent RNA sets. ** *p*<0.01; *** *p*<0.001. (B) The localization pattern of CRISPLD2 by immunohistochemistry in vehicle or P4-treated uteri. Uterine sections were collected from P4 or Veh treated wild-type (a and b) and PRKO (c and d) mice for 6 hours.

P4 regulates implantation, decidualization, and glandular development *via* a complex paracrine signaling network [32], [35]–[37]. Endometriosis has been associated with a reduced response to P4 in both the eutopic and ectopic endometrium. To determine whether *Crispld*2 is a P4 and PR target gene, we performed real-time PCR in the uterine samples of ovariectomized wild-type and PRKO female mice treated with vehicle (sesame oil) or P4 for 6 hours. As shown in Fig. 6A, mRNA transcript of *Crispld*2 was significantly increased in the uteri of P4 treated wild-type mice compared to vehicle treated wild-type mice. To investigate whether the P4 regulation of *Crispld*2 is relevant to PR, we examined the expression of *Crispld*2 in the uteri of wild-type and PRKO mice. After P4 treatment, induction of *Crispld2* was not detected in the PRKO mice (Fig. 6A).

To analyze the spatial expression of CRISPLD2 by P4 in the uterus, we performed immunohistochemistry in the vehicle or P4-treated wild-type and PRKO mice. Consistent with the real-time PCR outcomes, we observed CRISPLD2 signal in the glandular and luminal epithelium of the uterine section obtained from P4-treated wild-type uterus (Fig. 6B). The CRISPLD2 signals were not detected in the vehicle treated wild-type and vehicle or P4 treated PRKO uterine sections. These results indicate that CRISPLD2 expression is regulated by P4 and PR in uterus.

### E2 induces *Crispld2* expression in the murine uterus

**Figure 7:**
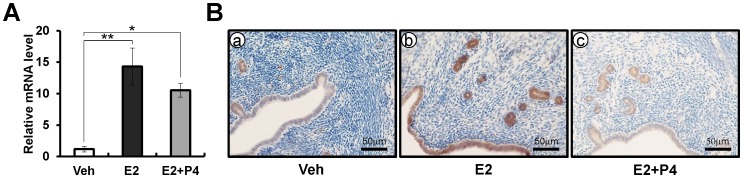
The expression of CRISPLD2 by steroid hormones. (A) The expression level of *CRISPLD2* from vehicle, or estrogen (E2) treated uteri by real-time PCR. Total RNA used for the real-time PCR assays was prepared from ovariectomized wild-type C57BL/6 mice treated with vehicle, E2, or E2+P4 for 6 hours. The results represent the mean ± SEM of three independent RNA sets. * *p*<0.5; ** *p*<0.01. (B) The localization pattern of CRISPLD2 by immunohistochemistry in vehicle, E2, or E2+P4 treated wild-type mice uteri for 6 hours. Nuclei were counterstained with hematoxylin.

As endometriosis is an E2-dependent disease, E2 regulation of CRISPLD2 was investigated. Ovariectomized female mice were treated with vehicle (sesame oil), E2 (0.1 mg/mouse), or E2 plus P4 (1 mg/mouse) for 6 hours. Our real time PCR results show that *Crispld2* mRNA expression was significantly increased in the uteri of mice treated with E2 or E2+P4 as compared with vehicle. Furthermore, immunohistochemical analysis that CRISPLD2 was highly expressed in both luminal and glandular epithelial cells of the uteri treated with E2 or E2+P4 for 6 hours as compared with vehicle (Fig. 7). These results suggest that the expression of CRISPLD2 was regulated by E2 as well as P4.

## Discussion

PR is a major mediator of epithelial-stromal cross talk through inhibition of the mitogenic effects of E2 and events leading to the disruption of this communication can lead to P4 resistance in the uterus [38]–[41]. P4 resistance is seen in a wide variety of diseases including infertility, endometriosis, and endometrial carcinoma [35], [36], [42]. Thus, the identification of P4-PR regulated genes is crucial for understanding the causes of impairments in proper uterine function. Herein, we show that expression of CRISPLD2 is altered in the uterus of endometriosis patients, and CRISPLD2 is consistently expressed during pregnancy *via* P4-PR signaling.

P4-PR signaling is critical during implantation, decidualization, and glandular development. *Crispld2* was identified as a P4 target gene by using high density DNA microarray analysis [43]. However, the function of *Crispld2* has not been studied in endometriosis and uterine biology. Our results suggest that *Crispld2* is differentially expressed by the epithelial cells as well as stromal cells of the murine uterus. During the pre-implantation stage in mice, CRISPLD2 can only be detected in then glandular epithelium. However, during implantation and post-implantation stages, CRISPLD2 can also be observed in stromal cells and decidual cells. These results suggest that CRISPLD2 may have an important role in the uterine epithelium during pre-implantation stage as well as for stromal cell decidualization at during the implantation and post-implantation stages. Our results show that the expression patterns of CRISPLD2 and PR/STAT3 are highly correlated in decidual cells. These results suggest that CRISPLD2 may mediate PR/STAT3 function during decidualization.

Endometriosis is associated with aberrant gene expression in the eutopic endometrium of women with disease. Aberrant gene expression of critical factors such as aromatase, endometrial bleeding factor, leukaemia-inhibitory factor, matrix metalloproteinases, and progesterone receptors, are dysregulated in the endometrium of women with endometriosis [36], [44]–[48]. Progesterone resistance is a hallmark of ectopic implants and plays a role in endometrium dysfunction associated with the endometriosis [2], [11]. We observed that the expression level of CRISPLD2 was significantly reduced in the endometrium from women with endometriosis (Fig. 2). This result suggests that CRISPLD2 may be involved in the pathogenesis or clinical sequelae of endometriosis. CRISPLD2 was expressed largely in endometrial stromal cells, and the expression was spatially and temporally regulated throughout the menstrual cycle (Fig. 1). Furthermore, we analyzed the expression pattern of CRISPLD2 during *in vitro* decidualization using hESC [31]. The level of CRISPLD2 was remarkably increased during decidualization process (Fig. 4 C and D). The expression profile of CRISPLD2 suggests that this molecule plays a role in decidualization. hESCs from patients with endometriosis show impaired decidualization [34]. Additionally, PRKO mice demonstrate a decidualization defect, suggesting that P4-PR signaling has a critical role during decidualization [32]. Previous studies have shown that P4 activates Indian Hedgehog (Ihh) signaling to induce expression of chicken ovalbumin upstream promoter transcription factor II (COUP-TFII) during decidualization in endometrial stromal cells [49], [50]. COUP-TFII has been shown to promote stromal cell decidualization via induction of bone morphogenetic protein 2 (BMP2) and inhibition of ERα activation [49]. STAT3 and PR crosstalk is required for successful embryo implantation and stromal cell decidualization in mice [51], [52]. Therefore, defining the specific role of CRISPLD2 would be requisite to further understand dysregulation of PR signaling pathways on etiology and infertility effect of endometriosis.

A key difference in women with endometriosis appears to be that retrograde menstruation alters the inflammatory response and immunosurveillance within the peritoneal fluid, facilitating proliferation and recurrence of new disease [27], [53], [54]. In affected women, maladaptive inflammatory responses are seen both systemically and within the eutopic endometrium, which is populated by inflammatory leukocytes. It is well recognized that chronic inflammation is associated with endometriosis as well as many other diseases. [28]. Endometriosis, however is a uniquely estrogen dependent inflammatory condition, associated with elevated tissue, peripheral and peritoneal cytokines levels [55]. An inflammatory survival cytokine required for decidualization, STAT3 is known to interact with PR, a molecule required for decidualization and known to interact with PR as well as the IL6 receptor in the uterus. Indeed, prolonged interaction with the IL6 causes [52], [56], [57]. In support of this inflammatory hypothesis, many investigators have documented elevated IL6 levels in the peritoneal fluid [58], [59] and serum of women with endometriosis [60], [61]. P4 has recently been shown to mediates the balance between anti-inflammatory and proinflammatory processes within uterus [62], [63] and P4 resistance is now becoming evident in a wide variety of diseases, including endometriosis. Herein, we showed that expression of CRISPLD2 was altered in the uterus of endometriosis patients, and CRISPLD2 expression was regulated by P4-PR signaling. Therefore, attenuation of an anti-inflammatory response gene such as *CRISPLD2* by progesterone resistance may alter the innate immune system and play important etiological role of endometriosis.

We examined the expression profile of *Crispld2* in mouse uteri during early pregnancy to study the role of *Crispld2* in female reproduction. The expression of *Crispld2* was gradually increased until 7.5 dpc. It is highly expressed in the glandular epithelium but at lower levels in the stromal cells at 3.5 dpc and then strongly expressed in the epithelial and stromal cells at 4.5 dpc. These pre-implantation time points are the period during which the uterine epithelium prepares for and permits the embryo to implant, suggesting that increased *Crispld2* in the epithelium may play a critical role in promoting implantation. CRISPLD2 was highly expressed in secondary decidual zone at 7.5 dpc (Fig. 5). However, CRISPLD2 was detected lower at 5.5 dpc than other stages. To better understand the role of CRISPLD2 during early pregnancy, the regulation mechanism of CRISPLD2 should be further investigated. These results suggest that CRISPLD2 may have an important role in epithelium at pre-implantation stage as well as stromal cells for decidualization at implantation and post-implantation stages.

We observed different expression patterns of CRISPLD2 between mice and women. The expression of CRISPLD2 was strongly detected both stromal and epithelial cells in secretory phase women. In mice, CRISPLD2 was detected in epithelial cells at pre-implantation stage and ovariectomized mice, while stromal cells were negative before decidualization occurs. The short reproductive cycle length observed in rodents, (aka: estrous cycle), makes them an ideal animal model for investigation of the reproductive cycle. However, mice do not have the cyclic spontaneous decidualization seen in humans. Therefore, we examined the expression profiles of CRISPLD2 in murine uterus during early pregnancy as well. CRISPLD2 is expressed by the epithelial cells at pre-implantation stage in mouse, while it is not detected at proliferative phase of human (a time of low P4 levels). However, CRISPLD2 was strongly detected in decidual cells of murine uterus (Fig. 5) as well as human endometrium from late secretory phase women, a time of high P4 levels (Fig. 1). Furthermore, CRISPLD2 increased during *in vitro* decidualization of hESCs (Fig. 4). To further investigate the expression of CRISPLD2 throughout pregnancy, we performed immunohistochemical analysis on uteri of C57BL/6 female mice at 9.5, 11.5, and 13.5 dpc. CRISPLD2 was only weakly expressed after 9.5 dpc (data not shown). Surprisingly, CRISPLD2 was strongly expressed in decidual cells of mouse as in human. These results suggest that CRISPLD2 may play an important role for decidualization in both mouse and human.

CRISPLD2 is a secreted glycoprotein with a conserved N-terminal secretory signal peptide [64]. In our results, non-secreted CRISPLD2 as well as secreted form were increased during *in vitro* decidualization of hESCs (Fig. 4D). However, we were unable to detect the secreted CRISPLD2 protein within the conditioned medium of hESCs being decidualized *in vitro* (data not shown). The ability of ovarian steroids to regulate uterine cell proliferation is highly dependent upon the growth factor communication networks linking the uterine stromal and epithelial cells. E2 is known to stimulate proliferation in uterine epithelial cells while P4 inhibits this stimulation. P4 achieves this inhibition by coordinating stromal-epithelial cross-talk [37], [65], [66] but the precise mechanisms is still unclear. CRISPLD2 has previously been shown to mediate distinct mesenchymal-epithelial signaling interactions in early compared with late fetal lung organogenesis [64]. CRISPLD2 expression in the cellular compartments in this model is under tight temporal and endocrine control, similar to what we see in the uterus. Of note, the mouse CRISPLD2 protein has 77% similarity with the human amino acid sequence and 92% similarity with rat, which may explain the differential expression patterns seen in mouse vs human. Therefore, CRISPLD2 may mediate stromal-epithelial cross-talk in the uterus. P4 is a crucial steroid hormone in the female reproductive system associated with the establishment and maintenance of pregnancy [40], [41], [67].

P4 regulation could be accomplished through progesterone receptors (PRs). The PRs are constituted with two isoforms, PRA and PRB, which are produced from the single gene containing alternative translation initiation site. In 1995 and 1996, Lydon, *et al.* investigated the P4-regulated pathways by using the mouse model carrying a null mutation of the progesterone receptor (PRKO) [32], [68]. Previous studies demonstrated the essential roles of P4-PR signaling for successful pregnancy [32], [68]. Resistance of P4-PR signaling triggers a variety of diseases in endometrium of women such as infertility, endometriosis, and endometrial carcinoma [35], [36], [42], [69]. In our study, the expression level of *Crispld2* was significantly increased by P4 in wild-type mice, but not in PRKO (Fig. 6). Both P4 and E2 are important for the maintenance of the female reproductive system and implantation. Especially, P4 is required throughout pregnancy. Embryo implantation depends on hormone regulation and endometrial status that are the proliferation of stromal cells and the differentiation of these cells into a decidual phenotype to prepare for blastocyst attachment. In mice in uterine sections of early pregnancy, the CRISPLD2 expression is strong in the epithelial cells of the pre-implantation uterus and then in decidual cells following implantation. These results suggest that CRISPLD2 may play an important during implantation and decidualization as a PR target.

The binding of P4 to PR results in PR nuclear translocation and subsequent alteration of P4 target gene transcription. Recently, Rubel et al. identified uterine PR-regulated mechanisms and downstream targets via chromatin immunoprecipitation followed by deep sequencing (ChIP-Seq) [70]. This study provided an important dataset for identification of target genes in the uterus regulated by the P4-PR. These ChIP-Seq results showed that PR directly binds to the proximal promoter region of *Crispld2* gene in the uteri of ovariectomized mice following treatment with P4. Furthermore, our study herein has shown that CRISPLD2 was significantly increased both epithelial and stromal compartments of the human endometrium during the secretory phase as compared to proliferative phase (Fig. 1), a time of elevated P4 levels and the onset of decidualization [71]. This further supports a role for P4-PR in CRISPLD2 regulation in endometrium of both mice and humans. Additionally, the expression of CRISPLD2 is upregulated by glucocorticoid receptors on cultured airway smooth muscle cells [72] and upregulated by retinoic acid in myeloblastic leukemia cells (Gene Expression Omnibus (GEO) database GDS1215). PR, glucocorticoid receptors, and retinoic acid signaling pathways are relevant to uterine physiology and to the pathogenesis of endometriosis. Although ligand binding elicits distinct hormone-specific responses, PR and GR recognize identical DNA response elements [73]. A question that continues to engage the steroid receptor field is how these transcription factors achieve DNA target specificity despite this degeneracy. Therefore, the exact molecular mechanism of this regulation requires further study.

In summary, this study observed that dysregulation of CRISPLD2 in endometrium from women with endometriosis. CRISPLD2 is highly expressed during decidualization in both mouse and human. We determined that CRISPLD2 is a one of target gene regulated by P4-PR response. These results suggest the P4 regulation of CRISPLD2 may play an important role for uterine function and pathology of endometriosis.
